# Effects of probiotic supplement Lactobacillus Plantarum CECT7485 and Lactobacillus Brevis CECT7480 on sleep quality in patients with anxiety and depression comorbidity

**DOI:** 10.1192/j.eurpsy.2023.976

**Published:** 2023-07-19

**Authors:** Y. Denysov, G. Putyatin, S. Moroz, V. Semenikhina

**Affiliations:** 1 Psychiatry, psychotherapy, addictology and medical psychology, Donetsk national medical university, Kropyvnitskyi; 2 Psychiatry, Municipal Enterprise “Dnipro Multiprofile Clinical Hospital for the Provision of Psychiaric Care of Dnipropetrovsk Regional Council”, Dnipro, Ukraine

## Abstract

**Introduction:**

Recent studies have supported that Lactobacillus plantarum can reduce the severity of anxiety and depression. However, previous studies did not focus on the sleep quality. This study determines whether Lactobacillus Plantarum CECT7485 and Lactobacillus Brevis CECT7480 reduce the severity of insomnia, and improves sleep quality in patients who comorbidity of depression and anxiety disorders.

**Objectives:**

An assessment of insomniac effects a probiotic supplement containing Lactobacillus Plantarum CECT7485 and Lactobacillus Brevis CECT7480 (PLANTARUM) in patients with anxiety and depression comorbidity undergoing treatment with selective serotonin reuptake inhibitors (SSRI) antidepressants.

**Methods:**

Sixty patients with mixed anxiety and depressive disorder (according to ICD-10 diagnostic criteria F41.2) were included in an 8-week open label study. Thirty participants received either SSRI antidepressants with PLANTARUM at a dose of 1.0 × 109 CFU once per day and thirty patients received SSRI antidepressants only. The severity of insomnia was assessed using Insomnia Severity Index (ISI). The severity of depressive symptoms was rated using Hamilton Depressive Rating Scale (HDRS). The severity of anxiety symptoms was assessed using Hamilton Anxiety Rating Scale (HAM-A) and General Anxiety Disorder Scale (GAD-7).

**Results:**

After 8 weeks intervention, a significant reduction of ISI total score (from 22,1±2,8 to 14,1±2,1) was detected in patients with anxiety and depression who prescribed SSRI antidepressants and PLANTARUM (p˂0,05), compared with participants who didn’t receive probiotics (p>0,05). Also, we detected a significant improve sleep quality of insomniac patients with comorbidity of anxiety and depressive symptoms (p˂0,05) who received SSRI antidepressants and probiotic supplement Lactobacillus Plantarum CECT7485/Lactobacillus Brevis CECT7480.

**Conclusions:**

The present data illustrated that probiotic supplement Lactobacillus Plantarum CECT7485 and Lactobacillus Brevis CECT7480 is a feasible for adjunctive to SSRI antidepressants intervention for insomniac patients with anxiety and depressive comorbidity

**Disclosure of Interest:**

None Declared

